# Learning From the Past: The Role of Social and Behavior Change Programming in Public Health Emergencies

**DOI:** 10.9745/GHSP-D-22-00026

**Published:** 2022-08-30

**Authors:** Martha Silva, Paula Tallman, Jeni Stolow, Rachel Yavinsky, Julia Fleckman, Kamden Hoffmann

**Affiliations:** a Tulane University School of Public Health and Tropical Medicine, New Orleans, LA, USA.; b Loyola University Department of Anthropology, Chicago, IL, USA.; c Population Reference Bureau, Washington, DC, USA.; d MOMENTUM Integrated Health Resilience, IMA World Health, Washington, DC, USA.

## Abstract

The contributions of social and behavior change research/programming in 6 recent epidemics highlight the importance of further integrating such expertise into outbreak response.

## Key Messages

- The prevalence of emerging infectious diseases (EIDs) is rising at an unprecedented rate, and influencing human behavior is often the first line of defense to slow disease transmissions.
- We synthesize the contributions that social and behavior change research and programming has made in 6 recent EIDs that reached epidemic proportions: HIV, severe acute respiratory syndrome, Middle East respiratory syndrome, Zika virus, Ebola virus disease, and coronavirus disease.
- Analysis of successful and unsuccessful responses to EID outbreaks suggests 5 primary lessons learned that can be used by SBC experts in future EID responses: engage communities, build trust through transparent risk communication, segment audiences for tailored interventions, prioritize behaviors, and cultivate political will and commitment.
- Incorporating SBC experts into EID responses can reduce mortality and improve efficiency in such precarious and time-sensitive settings.

## INTRODUCTION

The prevalence of emerging infectious diseases (EIDs) is rising at an unprecedented rate as a result of globalization, deforestation, economic growth, increased human mobility, urbanization, and fragile health systems.^1^^–^^5^ When new diseases emerge, medical and public health practitioners quickly begin to evaluate strategies to mitigate disease transmission and to use clinical interventions that can prevent or treat the emerging disease.^6^ However, behavioral responses—and the systems and structures that shape behavior—are often the first line of defense. As Dr. Deborah Birx stated during a press briefing on the coronavirus disease (COVID-19) response in March 2021, “there’s no magic bullet … it’s just behaviors.”^7^

Social and behavior change (SBC) refers to activities or interventions that examine and facilitate behavior change and the social and environmental factors that drive them.^8^ This includes behaviors leading to the prevention of disease transmission and those related to the promotion of health and well-being, as well as actions addressing structural and institutional environments. SBC contributions to EID preparedness, response, and recovery are distinct.^9^ Here, we focus on public health emergency response, in the context of which SBC experts develop approaches to reduce disease transmission under difficult and rapidly evolving circumstances.

Encouragingly, there have been notable advances in including SBC strategic approaches at the outset of public health emergency responses. In the International Health Regulations 2005 and Joint External Evaluation Tool, risk communication and community engagement are central to the World Health Organization guidance around communication in emergencies,^10^ and the U.S. Agency for International Development included SBC communication as 1 of 3 response pillars during the recent Zika outbreak in the Americas.^11^ However, despite these contributions, SBC experts are still not consistently included in outbreak response teams.^12^ This is due to limitations in human and financial resources and an “evidence-gap” regarding how SBC and other social sciences can improve epidemic responses, their impact, and their cost-effectiveness.^13^^,^^14^

The COVID-19 global pandemic presents an opportunity to revisit EID responses.^15^ Therefore, now is the time to emphasize the value of SBC and advocate for its inclusion as central to the global health emergency response ecosystem going forward. We synthesize the contributions that SBC research and programming have made in the emergency responses to 6 of the most recent EIDs that reached epidemic proportions: HIV, severe acute respiratory syndrome (SARS), Middle East respiratory syndrome (MERS), Zika virus (ZIKV), Ebola virus disease, and COVID-19 (Table 1). Based on key examples of successes and failures in recent EIDs, we synthesize 5 lessons learned that are grounded in past real-world events, yet applicable to present and future epidemics.

**TABLE 1.** Recent Emerging Infectious Diseases That Have Become Epidemics and Pandemics

**HIV/AIDS**	**Emergence**: The HIV/AIDS epidemic began in the 1970s, spreading from central Africa to New York and then around the world. **Health effects**: HIV infection is currently considered a chronic condition manageable with medication.^16^ **Global magnitude**: Approximately 36.3 million people worldwide have died of AIDS-related complications to date.^17^
**EVD**	**Emergence**: The West African EVD outbreak first emerged in December 2013 in Guinea. The outbreak quickly spread to neighboring countries, such as Liberia and Sierra Leone, and eventually spread to 7 more countries before ending in 2016.^18^ **Health effects**: EVD has an average case fatality rate of 50%.^19^ **Global magnitude**: This outbreak of EVD resulted in over 28,600 cases and 11,325 deaths.^19^
**ZIKV**	**Emergence**: The ZIKV outbreak began in 2015 in Brazil and soon spread throughout the Americas. **Health effects**: ZIKV infection during pregnancy is associated with preterm birth and miscarriage and can cause infants to be born with congenital malformations. Zika can also cause neurologic complications in adults, including neuropathy and myelitis.^20^ **Global magnitude**: To date, a total of 86 countries and territories have reported evidence of ZIKV infection.^20^
**SARS**	**Emergence**: SARS first emerged in Foshan, China, in November 2002^21^ and spread to 4 other countries. **Health effects**: SARS can result in acute lung injury, respiratory distress, and death, with a global case fatality ratio of 11%.^22^ **Global magnitude**: The SARS epidemic led to 8,422 probable cases and 916 deaths.^23^
**MERS**	**Emergence**: MERS first emerged in Saudi Arabia in 2012 and spread to 27 countries over the next 2 years.^24^ **Health effects**: MERS has a high case-fatality ratio (34.4%), which generated substantial global panic.^24^ **Global magnitude**: Only 2,574 cases of MERS have been laboratory-confirmed around the world.
**COVID-19**	**Emergence**: The novel COVID-19 began in Wuhan, China, in late 2019 and spread to almost every country globally. **Health effects**: COVID-19 has an average case fatality rate of approximately 2%^25^ and can cause long-term morbidities following infection.^26^ **Global magnitude**: As of June 2022, this pandemic has killed more than 6 million people.^25^

Abbreviations: COVID-19, coronavirus disease; EVD, Ebola virus disease; MERS, Middle East respiratory syndrome; SARS, severe acute respiratory syndrome; ZIKV, Zika virus.

Now is the time to emphasize the value of SBC and advocate for its inclusion as central to the global health emergency response ecosystem going forward.

## LESSON LEARNED #1: ENGAGE COMMUNITIES AS A KEY PILLAR OF EMERGENCY RESPONSES

Although the term “community engagement” is used widely across different contexts, concerning EIDs, it refers to the process of working collaboratively through community groups and using dialogue to establish trust with a community in the face of an outbreak.^27^ In the area of voluntary family planning, community group engagement approaches have been identified as a high-impact practice.^28^ Examples of community engagement activities include hosting local community meetings, recruiting community members for social mobilization activities, collaborating with religious leaders, and engaging with local journalists and radio for risk communication that considers the unique traits of the community.^29^ Effective community engagement emerged as a priority strategy during the West African Ebola epidemic of the last decade.

In Guinea, Liberia, and Sierra Leone, past wars, ethnic tensions, and the legacy of colonialism produced high levels of fear and hostility to national and international outbreak response teams working to stem the escalating death count during the summer of 2014.^27^^,^^30^^–^^32^ Unfortunately, in Guinea, 8 outbreak responders were murdered as a result of local suspicion and mistrust.^33^ A local police officer said the villagers believed that Ebola “is nothing more than an invention of white people to kill black people.”^34^ Within this environment of severe distrust, top-down externally driven responses were particularly inappropriate for local needs.^35^ Yet, at the beginning of the epidemic, emergency response teams recruited new and external community health workers, instead of engaging existing workers, community leaders, and influencers who were known and trusted in their communities.^32^

Eventually, the vital importance of “community engagement” became apparent as public health responders saw that programs prioritizing collective problem analysis, cocreation of solutions, tailored interventions, and local ownership of response were more effective than those that did not prioritize these elements.^31^^,^^36^^,^^37^ Community engagement elevated trust in the health system, which helped ensure effective responses to the crisis and was crucial in building more resilient health systems during this outbreak.^38^ Specifically, these approaches increased health facility response, built trust in the health system by communities, and improved communication efforts that ensured community members were treated as active participants during the response.^38^ In addition, health leadership at various levels recognized the need for health systems to be ready to prevent, prepare for, and respond to shocks and stresses presented by the outbreak. In a post-Ebola qualitative study in Liberia, participants mentioned the importance of “strengthening existing community institutions and relationships in calm, non-emergency settings,” with leadership from district health officers to formalize communication strategies by the health systems before health crises occur.^39^

As was the case during the onset of the Ebola outbreak in West Africa, early stages of the COVID-19 outbreak in populations such as Singapore’s migrant communities were plagued by a series of missteps in community engagement, leading to high infection rates and deep mistrust of leadership among these groups.^40^ Subsequent broad, 2-way engagement with a focus on agency, autonomy, and empowerment of communities has resulted in 90% of migrant workers in Singapore being vaccinated.^41^ With the availability of COVID-19 vaccines, calls to action for a community-engaged response have included establishing local community COVID-19 vaccine task forces to ensure bidirectional communication and deep understanding of factors likely to drive vaccine uptake.^41^

## LESSON LEARNED #2: BUILD TRUST THROUGH TRANSPARENT RISK COMMUNICATION

Alongside strategies such as community engagement, transparent risk communication—or open sharing of information as it is gathered—can help to build and maintain public trust in health institutions, public health authorities, and community leaders.^42^ At the outset of the SARS epidemic in 2002, local officials in China failed to communicate the gravity of the situation to the general public and the Chinese government waited 4 months to report the clusters of infections to the World Health Organization.^43^^,^^44^ In the interim, SARS spread around the globe, ultimately reaching 29 countries and causing 774 deaths.^45^

Amidst the global chaos caused by SARS, and in contrast to China’s initial response, Singapore was internationally praised for its transparent risk communication. This included building and maintaining trust with the public, sharing information openly, and encouraging public participation in public health action.^46^ For example, the Ministry of Health and officials from the Singaporean government held joint press briefings via traditional media where they acknowledged that they had little control over the external circumstances but were committed to sharing information in a timely yet informed manner.^47^ There were no time limits on the briefings and the practice of “speaking with one voice” via top health and government officials built trust and calmed public fears.^47^^,^^48^ Their transparency and willingness to publicly showcase the behaviors they were promoting, such as mask wearing, made the Singaporean government highly effective in communicating risks and supporting behavior change.

Unfortunately, the lessons learned in building and maintaining trust in public health responses were not initially taken up by all countries affected by MERS a decade later. For example, the early response to MERS by the South Korean government was heavily criticized for its opacity,^49^ as the government did not initially reveal the names of the hospitals treating MERS patients, creating substantial public distrust.^50^ Because of this, many South Koreans turned away from traditional media and to social media to gain information about this disease.^51^ The interpersonal nature of social media increased the likelihood that users believed their trusted online contacts’ information or opinions on MERS.^52^ Specifically, Yoo et al. found that receiving MERS-related information via social media networks was positively related to users’ “perceived threat” of MERS and to increased levels of intention to wash hands and practice cough etiquette.^53^ Another study found that social media stimulated the adoption of preventative behaviors such as mask wearing, handwashing, and contact avoidance.^54^ Thus, the MERS epidemic demonstrated that when the perceived credibility and trustworthiness of public health organizations is low, people turn to trusted sources in social media, and social media can substantially influence public health behaviors.^54^

The utility of social media as a risk communication channel remains undisputed; however, the most recent experiences with COVID-19 also highlight the potential for social media to contribute to an “infodemic”—an overabundance of information that can be hijacked by misinformation.^55^^,^^56^ Countries around the world have experienced the rampant spreading of fake news and false scientific claims via social media.^57^^,^^58^ Although all countries were challenged in reconciling risk communication with a lack of evidence at the beginning of the COVID-19 pandemic, some countries, such as Greece, committed themselves to transparent risk communication^59^; others, such as the United States, emerged as super-spreaders of misinformation as early mismanagement of risk communication eroded public trust. Furthermore, despite evidence for transmission of COVID-19 via respiratory droplets, the U.S. Surgeon General used Twitter to encourage the public not to buy masks, alluding they were not an effective prevention method for the general population.^60^ It later became clear that this messaging was driven by shortages in personal protective equipment and the need to keep available stocks for medical personnel. Thus, a lack of transparency produced conflicting messaging, substantial confusion, and generalized mistrust in government responsiveness.

## LESSON LEARNED #3: SEGMENT AUDIENCES FOR TAILORED INTERVENTIONS

Audience segmentation and tailored interventions help meet the needs of specific types of target audiences based on demographic or psychometric characteristics,^61^ such as attitudes, perceptions, beliefs, concerns, and information needs.^62^ Audience segmentation has been important in combating the HIV epidemic by understanding at-risk and impacted populations and ensuring that public health messaging and interventions reflect their unique circumstances.^63^

Audience segmentation has been important in combating the HIV epidemic by understanding at-risk and impacted populations and ensuring that public health messaging and interventions reflect their unique circumstances.

Despite major advances in HIV prevention and treatments,^64^ disparities in HIV infection and related poor health outcomes across different populations remain. For example, in the United States, gay and bisexual men are the group most severely affected, with young, Black, gay, and bisexual men being the only population in which new HIV infections have recently increased.^65^ These groups face unique barriers to HIV prevention and care including racism, homophobia, HIV stigma, and medical mistrust,^66^^,^^67^ all of which can lead to their unwillingness to engage with health services.

HIV is unique among recent EIDs in terms of timing, resources, and SBC involvement over long periods of time. However, it still provides an important example for informing behavioral responses to contemporary EIDs.^68^ For example, SBC experts know that social networks play a key role in promoting successful SBC interventions as people often seek friends’ advice to identify strategies to reduce the risk of contracting HIV.^69^^,^^70^ One such intervention is where trusted and well-revered individuals in the community, known as popular opinion leaders (POLs),^71^ are recruited and trained to have conversations with friends about reducing the risk of contracting HIV.^72^ To connect with specific communities, such as young, Black, gay, and bisexual men, POL training is tailored to reflect the unique hurdles this group faces.^73^ In this way, POL interventions rely on solutions driven by community perceptions and beliefs and delivered by trusted leaders to increase prioritized health-promoting behaviors and practices among well-defined or segmented target populations.

Imbalanced audience segmentation and tailoring, however, can lead to stigmatized, biased, and gendered health responses. For example, the discovery that ZIKV could be sexually and vertically transmitted resulted in gender-exploitative behavioral recommendations that disproportionately impacted women, ignored the sociocultural context, and promoted infeasible behaviors.^74^^,^^75^ For instance, the government of Jamaica was one of several countries recommending that women avoid pregnancy for at least 18 months until the ZIKV epidemic subsided.^76^ These early communications assumed female reproductive autonomy and ignored structural barriers that still exist within the region, largely leaving men out of prevention messaging.^77^^,^^78^

Audience segmentation and tailoring approaches continue to be used in the global response to COVID-19, specifically using knowledge about “behavioral typologies” to inform health campaigns and vaccination uptake. Behavioral typologies refer to the types of behaviors that characterize particular subgroups. For example, in the United States, behavioral typologies related to COVID-19 range from “worried social distancers” to “uninformed skeptics.”^79^ Globally, public health organizations such as FHI 360 have outlined a strategy to use psychometric properties to distinguish people and populations for COVID-19 vaccine uptake. For example, “easy sells” are those who place a high amount of trust in health care providers but lack awareness of vaccine availability, and “active resistors” are those with personal, cultural, or religious antivaccine beliefs.^80^ FHI 360 uses this knowledge in health campaigns to address key perceptual and behavioral structures that influence intent to get vaccinated.^81^

## LESSON LEARNED #4: PRIORITIZE BEHAVIORS

Information overload has been a common element at the beginning of several epidemics, which can result in conflicting and confusing messages about preventative behaviors. Behavioral prioritization is the process through which consensus is reached among key stakeholders to prioritize the behaviors with the most potential to prevent EID transmission.^82^ For example, the Liberian government implemented a “firehose approach” at the beginning of the Ebola epidemic and encouraged a barrage of preventive behaviors, many of which were not directly linked to Ebola.^30^ Similarly, the early response to the ZIKV outbreak was challenged by a dearth of scientific knowledge about the disease’s evolved transmission routes and health consequences.^83^

ZIKV was known to spread via the *Aedes aegypti* mosquito and to typically present similarly to other arboviruses such as dengue, chikungunya, and yellow fever. Based on this, early responses recycled ineffective vector control campaigns and practices used for other flavivirus outbreaks.^84^ The discovery of ZIKV’s sexual transmission and unique health consequences, such as congenital Zika syndrome, contributed to an outpour of conflicting, inconsistent, and confusing prevention campaigns across the region. For example, in the first year of the U.S. Agency for International Development’s response to ZIKV, more than 30 variations of preventive behaviors were promoted.^85^ By 2018, behavior prioritization conducted at the regional level helped reduce the recommendations to a total of 7 behaviors, which were prioritized through a participatory process among organizations implementing Zika responses.^82^

Despite ample reflection on the importance of behavioral prioritization during ZIKV in Latin America, the communication of priority behaviors for COVID-19 was not always coherent or evidence-based. For example, while social distancing and stay-at-home recommendations were implemented in the United States, there was confusion regarding the need for disinfection and masks.^86^ Regarding the former, early in the pandemic, the World Health Organization and health agencies around the world recommended that people clean and disinfect surfaces.^87^ However, it emerged that COVID-19 transmission by fomites (inanimate surfaces or objects) had been presumed on the basis of studies with little resemblance to real-life scenarios.^88^ Health agencies pivoted in their behavioral recommendations accordingly.^89^ This example highlights the importance of communicating new evidence as it becomes available while acknowledging the impact it may have on reprioritizing behaviors.

## LESSONS LEARNED #5: CULTIVATE POLITICAL WILL AND COMMITMENT

As Stein stated succinctly^89^:

*Social and economic factors together with political will and commitment are increasingly recognized as being at least as influential as biological and technical feasibility in preventing, controlling and eradicating infectious disease outbreaks.*

We observed that across all recent EIDs, behavior change has been key to protecting the community’s, households’, and individuals’ health. Yet many of the SBC response “failures” we highlight here were due to lack of political will and support. For example, the mishandling of the SARS and MERS epidemics was partially attributable to a lack of political will on behalf of the Chinese and South Korean governments, respectively.^44^^,^^90^

The crucial role of political will in EID responsiveness has also been glaringly apparent in the global response to COVID-19. For example, in the United States, leadership consistently downplayed the threat posed by COVID-19 early in the pandemic^91^ and undercut and contradicted messages being relayed by public health experts.^92^ A slow U.S. federal response led to inadequate diagnostic testing and chaotic competition for limited supplies of personal protective equipment and ventilators^93^ and to the highest number of COVID-19-related deaths in the world.^94^ This outcome is undoubtedly, in part, due to a failure of U.S. policy and leadership.^95^

The crucial role of political will in EID responsiveness has also been glaringly apparent in the global response to COVID-19.

On the other hand, Greece successfully managed the first wave of COVID-19 because of evidence-based decision making by strong and decisive leadership, effective intragovernmental coordination, and transparent communication that supported high citizen compliance.^59^ These actions were driven by the knowledge that, after more than a decade of economic challenges, their health care systems could not handle an outbreak similar in magnitude to that in neighboring Italy.^96^ To communicate these risks, the government of Greece featured 2 leaders during COVID-19 press briefings, a professor focused on the medical developments and a secretary who communicated governmental decisions and crisis management measures.^97^ This approach resembled the way that Singaporean officials “spoke with one voice” during the SARS epidemic^48^ and contributed to similar public satisfaction with governmental leadership during these epidemics.^98^^,^^99^ These examples suggest that political will and commitments are foundational for the actions necessary to facilitate social and behavioral change.

## APPLYING LESSONS LEARNED TO FUTURE EID OUTBREAK RESPONSE

The examples we present of previous responses to EID outbreaks offer only a snapshot into recent history and are not intended to suggest that a one-size-fits-all strategic approach to public health emergencies is appropriate. Yet, these lessons learned can be operationalized for future responses to ensure the inclusion of SBC experts and other stakeholders who are prepared to navigate the complexities of behavioral change (Table 2).

**TABLE 2.** Operationalizing Social and Behavior Change Lessons Learned In Emergency Responses to Emerging Infectious Disease Epidemics

**Lessons Learned**	**Suggested Actions**	**Desired Outcome(s)**
Engage communities as a key pillar of emergency responses	Outbreak investigators should work with communities to: Conduct participatory action research to identify contextually relevant barriers to uptake of preventive behaviors Strengthen community-based networks for epidemiological and behavioral surveillance^100^ Support social mobilization for development and validation of risk-related communication strategies and messages	Communities are engaged in pandemic response, ensuring local ownership and leadership. Strategies and communications are appropriate for community audiences.
Build trust through transparent risk communication	Political leaders and public health spokespeople should: Communicate current state of affairs in a timely and consistent manner regarding known risks and spread of an emerging infectious disease Acknowledge gaps in knowledge and limitations, and reiterate commitment to communicating new evidence and its implications in a timely manner Strengthen existing community institutions in calm, nonemergency settings	Communities have trust in epidemic advisors and are more willing to follow advice and guidelines under changing circumstances.
Segment audiences for tailored interventions	Response coordinators should: Gather information and insights to understand the attitudes, perceptions, beliefs, concerns, and information needs of particular audiences, using the best social science evidence available Use tools such as FHI 360’s rapid audience assessment to support analysis and development of audience profiles^80^ Tailor interventions by designing strategies, tools, and materials that reflect audience profiles	Audiences receive messages that are fit to their needs and priorities. Messages are communicated in ways that respect community differences and do not promote stigma or biases.
Prioritize behaviors	Epidemic response teams should: Define criteria for behavior prioritization such as behaviors based on segmented audiences, the state of the evidence for each preventive behavior, and feasibility of adopting the behavior^85^ Harmonize risk communication messages and channels to “speak as a single voice”	Messages clearly communicate the most important behaviors for more effective interventions. Individuals understand the most important behaviors to undertake and are not overwhelmed with instructions or conflicting messages.
Cultivate political will and commitment	Public health leaders should: Proactively communicate with politicians and funders about the importance of social and behavior change expertise in preparedness and response Use honest conversations about past failures to inform future agendas Encourage multilateral coordination to promote efficiency and effectiveness Help high-income country leaders recognize the implications of outbreaks in low- and middle-income countries	Policy makers are willing to make clear and evidence-based decisions. Leaders prioritize effective pandemic response over short-term political motivations. Decision makers in high-income countries respond earlier to disease outbreaks in low- and middle-income countries.

These lessons must not be seen as siloed approaches or sequential in nature. They represent interconnected efforts that must be iteratively deployed through the ebbs and flows of a public health emergency. For example, the public’s trust in leadership and public services before a public health emergency is critical to response effectiveness, becoming more so when an EID is identified. The outset of an outbreak is a critical time when communication and behavioral adjustment are needed, even in the absence of definitive biological evidence about the EID, such as how it is transmitted, treated, and managed. Public health and government leaders need to quickly make recommendations for behavioral prioritization to thwart the disease progression (inferring, for example, from other diseases).

These lessons are interconnected efforts that must be iteratively deployed through the ebbs and flows of a public health emergency.

As more evidence arises, leaders must communicate updated evidence-based strategies with transparency. Early missteps in communication and engagement can lead to skepticism and severely erode public trust in the ability of health care providers and government leaders to navigate the challenges of the EID. As seen during the MERS epidemic in South Korea and during COVID-19 in the United States and elsewhere, trust in political and public health leaders can be difficult to regain once lost. Furthermore, as seen in the Ebola epidemic, mistrust can undermine the entire public health response. Indeed, as Jakovljevic stated^101^:

*distrust mentality, conspiracy thinking and blame games may have detrimental effects not just on the individual level, but on the level of the whole groups, communities and global world.*

As more information about an EID becomes available, targeted preventive behaviors have to be prioritized and health communication strategies must consider specific audiences to increase the probability that those behaviors will be adopted. In the case of COVID-19 in the United States, a range of factors contributed to creating several subgroups of vaccine-hesitant people. These factors include the government’s denial of the extent of the problem and lack of transparent communication, confusion regarding what behaviors were relevant, and a widespread “infodemic” of misinformation about COVID-19 (primarily spread via social media). As of January 2022, only 64% of the U.S. population was fully vaccinated,^102^ despite evidence that vaccines substantially lower the risk of dying from COVID-19.^103^

A key question in addressing vaccine hesitancy is whether mandates are an appropriate strategy. Indeed, mandates outlawing smoking in public areas and requiring seat belts, among others, have been highly effective in lowering morbidity and mortality.^104^ However, understanding the unique psychometric properties of vaccine-hesitant subgroups indicates that this may not be the best approach in all cases. According to Richwine^105^:

*[Vaccine-hesitancy] stems from deeply held convictions about bodily integrity and autonomy. Giving them a no-jab-no-job ultimatum causes them psychological distress. It sows resentment, distrust, and alienation. It stokes fears of a slippery slope. It causes real harm.*

In other words, vaccine mandates could increase aversion to public health interventions, reduce vaccination in such groups, and actually undermine the goals of COVID-19 vaccination campaigns.^106^ Alternatively, as seen in Singapore, community engagement with a focus on agency, autonomy, and empowerment of communities could potentially increase vaccinations in those previously hesitant.^40^

Thus, we are not advocating the application of lesson learned after lesson learned in a set timeline. Instead, it is the continuous and flexible deployment of SBC strategies in an evidence-based and holistic manner in conjunction with epidemiological and clinical interventions that can yield the greatest benefits for public health when all integrated within an EID response.

## CONCLUSION

Based on responses to COVID-19 and prior epidemics, we observe that SBC approaches—such as audience segmentation and tailoring, transparent risk communication, community engagement, and behavioral prioritization—integrally support public health. However, strategies to influence social and behavioral change cannot be leveraged without political will and commitment. In the last few years, researchers have increasingly pointed this out and advocated for the inclusion of social science, including SBC experts, at the outset of EID responses.^13^ Additionally, several initiatives are using SBC to continue combatting the COVID-19 pandemic, including the COVID-19 Communication Network, READY Initiative, GOARN (Global Outbreak Alert and Response Network), and Collective Service.^107^^–^^110^ But more is needed to ensure that SBC receives attention and investments that are on par with allied fields such as virology, epidemiology, and clinical medicine. COVID-19 continues to evolve, and future EIDs will emerge. If further integrated into EID preparedness and responses, SBC experts can implement a proactive, multidimensional approach based on many of the lessons learned presented. This is why SBC experts must be an integral part of EID response.

## Notes

**Peer Reviewed**

**First published online:** August 5, 2022.
